# Impact of an abdominal belt on breathing patterns to improve the quality of whole-heart coronary magnetic resonance angiography: comparison between UK and Japan

**DOI:** 10.1186/1532-429X-13-S1-P230

**Published:** 2011-02-02

**Authors:** Masaki Ishida, Andreas Schuster, Shinichi Takase, Geraint Morton, Amedeo Chiribiri, Tobias Schaeffter, Hajime Sakuma, Eike Nagel

**Affiliations:** 1 King's College London Division of Imaging Sciences and Biomedical Engineering, London, UK; 2 Mie University Hospital, Tsu, Japan

## Introduction

Navigator techniques allow the patient to breath freely during a whole heart coronary magnetic resonance angiography (WHCMRA) scan. However, long measuring times, caused by the necessity to synchronize the cardiac and the breathing cycle, and complex motion patterns lead to the suboptimal image quality. To overcome this problem, an abdominal belt (BELT), which can suppress the abdominal breathing motion and, thus, improve WHCMRA image quality, has been suggested by a Japanese group1. However, its feasibility has never been shown for a Western population.

## Purposes

The purpose of this study was to investigate whether the abdominal belt has similar impact on breathing patterns in UK and Japanese patient populations.

## Methods

30 patients (15 British and 15 Japanese) were evaluated (Achieva, 1.5T, Philips Medical Systems). Five real time navigators were used to collect motion parameters: right and left diaphragmatic cranio-caudal (CC), right thoracic anterior-posterior (AP), right thoracic left-right (LR) and upper abdominal wall AP. Measurements were performed in the supine position with free breathing for one minute before and after a tight-fitting BELT wrapped around the patient’s abdomen. End expiratory position (EEP), end inspiratory position (EIP) and end expiratory duration (EED) for the right diaphragm, and respiratory rate (RR) were obtained. Scan efficiency was calculated from the duration within the 5mm gating window per minute and heart rate

## Results

**Figure 1:**
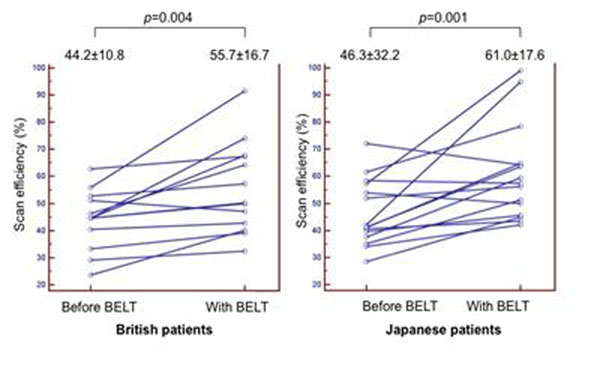
Scan efficiency before and with BELT in British and Japanese patients

Height and weight of British patients were significantly larger than in Japanese patients (171.2±10.8 cm vs 160.8±8.5 cm, p=0.007; 80.5±22.5 kg vs 59.9±7.7 kg, p=0.004). After fitting the BELT, EEP-EIP decreased (all patients, 14.9±6.2 mm to 9.4±3.8 mm, p<0.001; British patients, 15.9±6.0 mm to 9.7±3.1mm, p=0.001; Japanese patients, 14.0±6.4 mm to 9.1±4.6mm, p=0.001), RR increased (all patients, 10.0±3.1 Hz to 11.2±3.0 Hz, p=0.003; British patients, 9.5±2.8 Hz to 10.7±2.8 Hz, p=0.038; Japanese patients, 10.4±3.5 Hz to 11.8±3.1Hz, p=0.036), and calculated scan efficiency using 5mm gating window increased (all patients, 45.3±11.4 % to 58.6±17.0 %, p<0.001; British patients, 44.2±10.8 % to 55.7±16.7 %, p=0.004; Japanese patients, 46.3±32.2 % to 61.0±17.6 %, p=0.001) (Figure 1). No significant differences were found between British and Japanese patients both before and after application of the BELT.

## Conclusion

The current results indicated that the scan efficiency significantly increased by using the BELT both in British and Japanese patients and suggested that application of a BELT can improve WHCMRA image quality in a Western patient population similar to the Japanese results.

## References

[B1] Kato JACC 2010 56 98320828652

